# Efficient Sensor Node Selection for Observability Gramian Optimization

**DOI:** 10.3390/s23135961

**Published:** 2023-06-27

**Authors:** Keigo Yamada, Yasuo Sasaki, Takayuki Nagata, Kumi Nakai, Daisuke Tsubakino, Taku Nonomura

**Affiliations:** 1 The Department of Aerospace Engineering, Tohoku University, Sendai 9808579, Japan; 2 The Department of Aerospace Engineering, Nagoya University, Nagoya 4648603, Japan

**Keywords:** monitoring, state estimation, scheduling, optimization, sensor networks

## Abstract

Optimization approaches that determine sensitive sensor nodes in a large-scale, linear time-invariant, and discrete-time dynamical system are examined under the assumption of independent and identically distributed measurement noise. This study offers two novel selection algorithms, namely an approximate convex relaxation method with the Newton method and a gradient greedy method, and confirms the performance of the selection methods, including a convex relaxation method with semidefinite programming (SDP) and a pure greedy optimization method proposed in the previous studies. The matrix determinant of the observability Gramian was employed for the evaluations of the sensor subsets, while its gradient and Hessian were derived for the proposed methods. In the demonstration using numerical and real-world examples, the proposed approximate greedy method showed superiority in the run time when the sensor numbers were roughly the same as the dimensions of the latent system. The relaxation method with SDP is confirmed to be the most reasonable approach for a system with randomly generated matrices of higher dimensions. However, the degradation of the optimization results was also confirmed in the case of real-world datasets, while the pure greedy selection obtained the most stable optimization results.

## 1. Introduction

Collecting information from sensor measurements is often the only viable approach when estimating the internal state or hidden physical quantities. The optimization of sensor positions was intensively discussed in order to determine the most representative sensors and to reduce the resulting estimation error, such as when monitoring sensor networks [1,2,3,4], fluid flows around objects [5,6,7,8,9,10,11,12,13,14,15], plants and factories [16,17,18], infrastructures [19,20,21], circuits [22], and biological systems [23], estimating physical field [24,25,26,27], and localizing sources [28,29]. Recent advances in data science techniques have enabled us to extract reduced-order models from vastly large-scale measurements of complex phenomena [30,31,32,33,34,35,36,37,38,39]. Therefore, the optimized sensor measurement is gaining importance for the reconstruction of complex phenomena from sensor measurements based on the data-driven reduced-order models [40,41], as well as for model-free machine learning [42,43] and data assimilation [44,45,46].

The physical aspects of the phenomena are interpreted as state space models, while the assumed system representations are often nominal. Our study implements optimization frameworks for sensor node selections based on the criteria of expected estimation errors defined for these models. Sensor measurements in many nondynamical configurations are statistically described by the Fisher information matrix (FIM), which originated in the parameter sensitivity analysis [47,48]. This measure addresses the expected error ellipsoid of the estimation using instantaneous measurements, which corresponds to the inverse of the Cramer–Rao bound, see Chapter 8 of [49,50]. The combinatorial problem structure is transformed into small subproblems in a greedy formulation [40,41,51], of which, the solution quality is known to be ensured by the submodularity property in such optimization problems [52,53,54,55,56]. This property of the optimization problem helps to exploit other criteria from information theory, including entropy and various types of information distances [3,57,58]. Moreover, greedy selections are empirically confirmed to effectively work even in the absence of submodularity [51,59,60]. There are also other approaches that seek the global optimum by relaxing the integer constraints on the selection variable [50,61], or by employing proximal algorithms that form proximity operators [62,63,64].

The subset selection of sensor nodes for dynamical systems is similarly conducted based on the estimation error covariance, while the measurement quality evaluation tends to be more cumbersome. The observability Gramian is the counterpart of the FIM for linear time-invariant (LTI) representations with deterministic dynamics, as shown in [65]. The actuator node selection, which is a dual problem of the sensor node selection, is similarly obtained using the controllability Gramian [66,67]. It is worth emphasizing that a previous analysis used examples of random and regular networks of nodes of tens or hundreds due to the computational cost. For example, a previous study reported a tremendous increase in the computation time of the greedy method and the SDP relaxation method for a power grid system of such a size [66]. There are likely to be computational issues when the optimization objective is further extended to the observability for the nonlinear state space models [44,68,69], the error covariance of the Kalman filter [70,71,72,73,74], and the $H_{2}$ norm of an LTI system, which can be reduced through balanced truncation [75,76,77]. Accordingly, the main interest in this study is the applicability of the Gramian-based methods for the larger scale system constructed by the data-driven modeling methods.

The main claims of this paper are listed below:

*Novel selection methods proposed:* Two novel selection methods are proposed and implemented. Those two methods utilize the gradients of the objective functions while relaxing them to continuous functions. Computations in both methods are expected to be remarkably accelerated as compared to those of existing greedy or relaxation-based algorithms since the gradients of objective functions are mere quadratic forms of the dimensions of sensor candidates.

*Comparison using high-dimensional and low-rank systems:* A comparison of selection strategies is also provided in Section 4 to illustrate the characteristics of each selection method in terms of the execution time and the acquired optimization measure. This comparison also elaborates effective computational complexities of each selection method, which is distinct from the results of the previous study.

**Figure 1:**
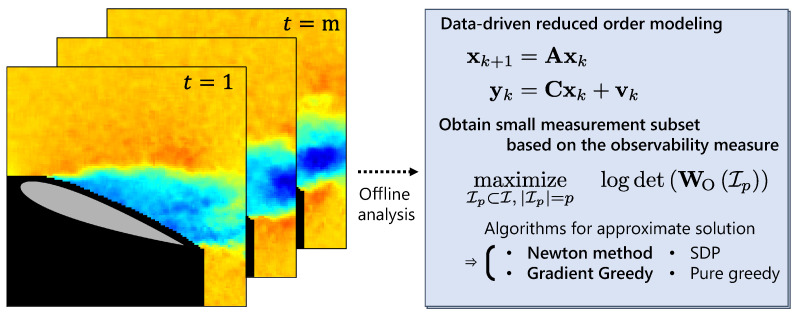
Brief description of this manuscript. (**Left**) The representative data point is revealed from “rich” measurement data by use of a data-driven model and optimization procedure. (**Right**) A data-driven method constructs linear reduced-order models before the optimization is conducted by approximate algorithms, including our novel methods, denoted by bold types.

Figure 1 depicts the main frame of this manuscript. In the following sections, the basic formulation of the optimization problem is revealed in Section 2 after providing the dynamical system of interest. Section 3 subsequently addresses our novel algorithms for optimization while the previously presented approaches are briefly included. The systems are constructed in two different ways, for comparison; one is constructed via random numbers as a general case in Section 4.1, and in the other case, a data-driven method using proper orthogonal decomposition (POD) [78] is applied to real-world datasets, as seen in Section 4.2 and Section 4.3

## 2. LTI State Space and the Observability Gramian

This section provides rudimentary formulations that represent dynamics and optimization tasks based on the observability of the system. Problem settings are first presented with basic equations while notations for our formulation are defined. The term “observability” is omitted hereafter unless it causes confusion with the controllability Gramian or other Gram matrices. One may be reminded that the methodologies presented here are similarly adopted to controllability optimization since these properties are dual for the linear systems. A discrete-time LTI state space model is considered with r(∈N) states and n(∈N) measurements. Let $\left(C,A\right)$ be an observable pair and an autonomous system

(1) $$x_{k+1}=Ax_{k}$$

(2) $$y_{k}=Cx_{k}+v_{k}$$

generates a trajectory of state variables $x_{k}\in R^{r}$ and observations $y_{k}\in R^{n}$. The subscript k∈N refers to a snapshot at the instance *k*. Assume the observation Equation (2) is corrupted by a Gaussian noise $v_{k}∼N\left(0|R\right)$, which is independent of the state $x_{k}$ and has the variance of the same amplitude, $E\left[v_{k}v_{k}^{⊤}\right]=R=\sigma^{2}I_{n}$, where $I_{n}$ is the identity matrix of size *n*. Here, $E\left[◯\right]$ is taking the expectation over the random variable. Some data-driven techniques systematically provide this linear representation from the measured data, such as proper orthogonal decomposition and dynamic mode decomposition [37,38]. In the state space representation obtained by the data-driven techniques, the state vector represents coordinates of a low-dimensional subspace inherent in the high-dimensional space of the measurement vector and, therefore, n≫r is assumed hereafter. The observability of the system is quantified by the Gramian equation

(3) $$\begin{array}{cc} \; & {\bar{W}}_{\;O}=\sum_{k=0}^{\infty }{\left(A^{⊤}\right)}^{k}C^{⊤}CA^{k}. \end{array}$$

In our formulation, this matrix is characterized by the linear least-squares estimation of the state vector that utilizes time-series measurements. The measurement equation is extended first, using from k=0 to k=l−1 of Equations (1) and (2):

(4) $$\begin{array}{ccc} y_{0:l-1} & := & {\left[y_{0}^{⊤},\;y_{1}^{⊤},\ldots ,y_{l-1}^{⊤}\right]}^{⊤}\\ & = & \left[\begin{array}{c} C\\ CA\\ \vdots \\ CA^{l-1} \end{array}\right]x_{0}+\left[\begin{array}{c} v_{0}\\ v_{1}\\ \vdots \\ v_{l-1} \end{array}\right]\\ & =: & C_{0:l-1}x_{0}+v_{0:l-1}, \end{array}$$

where $C_{0:l-1}$ and $v_{0:l-1}$ are the stacked components in the brackets in Equation (4). An estimate of $x_{0}$ is obtained by the linear inversion as follows:

(5) $$\begin{array}{cc} ∴\;{\tilde{x}}_{0} & ={\left(C_{0:l-1}\right)}^{†}y_{0:l-1}\\ \; & ={\left(C_{0:l-1}^{⊤}C_{0:l-1}\right)}^{-1}C_{0:l-1}^{⊤}y_{0:l-1}\\ \; & =x_{0}+{\left(C_{0:l-1}^{⊤}C_{0:l-1}\right)}^{-1}C_{0:l-1}^{⊤}v_{0:l-1}, \end{array}$$

where ${\tilde{x}}_{0}\in R^{r}$ and ${◯}^{†}$ stand for the estimate of $x_{0}$ and the Moore–Penrose pseudo inverse, respectively. The matrix $C_{0:l-1}$ is assumed to have full column rank due to n≫r. An error covariance matrix under the estimation ${\tilde{x}}_{0}$ is proportional to the inverse matrix of the Gramian, as shown in the following equation:

(6) $$\begin{array}{cc} \; & E\left[\left(x_{0}-{\tilde{x}}_{0}\right){\left(x_{0}-{\tilde{x}}_{0}\right)}^{⊤}\right]\\ \; & \propto {\left(C_{0:l-1}^{⊤}C_{0:l-1}\right)}^{-1}\\ \; & ={\left(\sum_{k=0}^{l-1}{\left(A^{⊤}\right)}^{k}C^{⊤}CA^{k}\right)}^{-1}\\ \; & \underset{l\to \infty }{⟶}{\left({\bar{W}}_{\;O}\right)}^{-1}. \end{array}$$

Here, let a small number of sensors be deployed while maintaining the quality of state estimation. The optimization problem is formulated as a selection of a subset $I_{p}=\left\{\;i_{1},\;\ldots ,\;i_{p}\right\}$ from all available *n* measurement nodes, labeled as $I_{n}=\left\{1,2,\ldots ,n\right\}$, where a measure of the Gramian is optimized under some constraints. A permutation matrix $P\left(I_{p}\right)$ that extracts a part of the measurement corresponding to the sensor indices $\left(I_{p}\right)$ is defined by

(7) $$\begin{array}{c} P\left(I_{p}\right)=\left[\begin{array}{c} e_{i_{1}}^{⊤}\\ \vdots \\ e_{i_{p}}^{⊤} \end{array}\right], \end{array}$$

where a unit vector $e_{i}\in R^{n}$ has unity in the *i*-th entry, and the rest is zero. The Gramian given by the selected sensors is rewritten as a subset function

(8) $$\begin{array}{cc} W_{\;O}\left(I_{p}\right) & =\sum_{k=0}^{\infty }{\left(A^{⊤}\right)}^{k}{\left(PC\right)}^{⊤}\left(PC\right)A^{k}, \end{array}$$

and, therefore, $W_{\;O}\left(I_{n}\right)={\bar{W}}_{\;O}$. Here, $W_{\;O}\left(I_{p}\right)$ can be calculated by solving the following Lyapunov equation:

(9) $$\begin{array}{cc} \; & A^{⊤}W_{\;O}\left(I_{p}\right)A-W_{\;O}\left(I_{p}\right)+{\left(PC\right)}^{⊤}\left(PC\right)=0,. \end{array}$$

Some measures for the optimization of $W_{\;O}\left(I_{p}\right)$ were presented in [66]. A maximization of the determinant of the Gramian is configured in the current study. The maximization problem is as follows:

(10) $$\begin{array}{cc} \; & \underset{I_{p}\subset I,\;\left|I_{p}\right|=p}{\mathrm{maximize}}\;\log\det\left(W_{\;O}\left(I_{p}\right)\right). \end{array}$$

The logarithmic form, which is monotone, is considered for the ease of calculations in the algorithms. This determinant maximization strategy is commonly used in the sensor placement and optimal design of interpolation methods [40,79].

## 3. Sensor Selection Strategies

The optimization problem (10) is a combinatorial problem and, therefore, finding the true optimum is computationally prohibitive. This section introduces four methods, as shown in Table 1, for obtaining a suboptimal yet reasonable solution to (10). Section 3.1 deals with convex relaxation approaches to (10), where the notations of *SDP* and *approximate convex relaxation* stand for the selection problems based on the semidefinite programming (SDP) of [67] and an approximate smooth convex relaxation for the Newton method, which is extended from [50], respectively. Subsequently, Section 3.2 provides formulations with regard to the *pure greedy* and *gradient greedy* methods, as a simplified greedy implementation of [66] for the matrix determinant maximization, while the latter is its linear approximation. The computational complexities are discussed in Section 3.3.

### 3.1. Convex Relaxation Methods

This strategy replaces the Gramian $W_{\;O}\left(I_{p}\right)$ by the weighted sum of the Gramian for every sensor candidate, namely the *relaxed Gramian. * Let the sum of the selection weight $s\in {\left[0,\;1\right]}^{n}$ be *p*, and the relaxed optimization problem of interest is:

(11) $$\begin{array}{c} \begin{array}{cc} \; & \underset{s}{\mathrm{maximize}}\;\log\det\left(Q\left(s\right)\right)\\ \; & \begin{array}{cc} \mathrm{subject to}\;\; & Q\left(s\right)=\sum_{i=1}^{n}s_{i}\;W_{\;O}\left(\{i\}\right),\\ \; & s_{i}\in \left[0,\;1\right],\;1^{⊤}s=p. \end{array} \end{array} \end{array}$$

This “weight” formulation is widely used in the various types of sensor selections, including linear inverse and Kalman filter estimations, [50,61]. The “gain” formulation, on the other hand, is employed to optimize the gains for yielding state variables from measurements. The group regularization is therein adopted to distinguish the representative measurement locations, which can also be found in the literature on sensor selection for linear inverse and Kalman filter estimations [62,63,64,80]. To the best of our knowledge, there is no existing study in which the gain formulation is applied to the sensor selection based on the observability Gramian. This may be because the Gramian is related to an infinite series of temporal measurements, and so are the gains for such measurements. Truncating Equation (6) to a finite time horizon is obviously a possible option, but dealing with a large solution vector and defining an appropriate finite time horizon as a hyperparameter are practically infeasible. Accordingly, the implementation of the gain formulation remains an interesting challenge.

#### 3.1.1. Semidefinite Programming-Based Selection (*SDP*)

Convex relaxation for Gramian optimization was previously introduced in the section II-B of [67]; therefore, the readers should refer to the original work for more details. It should be emphasized that an optimization problem in the discrete-time form is briefly revisited in our study and is included in Section 4 for a comparison of selection methods. An optimization problem (11) is transformed into the following SDP representation:

(12) $$\begin{array}{c} \begin{array}{cc} \; & \begin{array}{cc} \underset{s}{\mathrm{maximize}}\; & \log\det\left(Q\left(s\right)\right) \end{array}\\ \; & \begin{array}{cc} \mathrm{subject to}\;\; & s_{i}\in \left[0,\;1\right],\;1{\;}^{⊤}s=p,\;Q\left(s\right)⪰0,\\ \; & A^{⊤}Q\left(s\right)A-Q\left(s\right)+\sum_{i=1}^{n}s_{i}\;c_{i}^{⊤}c_{i}=0, \end{array} \end{array} \end{array}$$

where $c_{i}$ is the *i*-th row of the measurement matrix C, and 0 is a zero matrix with appropriate dimensions. It should be noted that the Lyapunov equation, imposed as a constraint in the problem, can be satisfied by SDP solvers introduced later. The complexity in Table 1 is based on a path-following method for a general primal–dual interior point method [81].

#### 3.1.2. Newton Method for Approximate Convex Relaxation, and Its Customized Algorithm with Randomized Subspace Sampling (*Approximate Convex Relaxation*)

This novel method solves the convex relaxation problem by applying the Newton method and a customized randomization technique applied to Equation (11), with a penalty term that bounds the weight variables added.

The description of the proposed method starts with the extension of the formulation of sensor selection for static systems first introduced in [50].

In their approach, the Newton method solved a weight formulation of a determinant optimization for the FIM of the linear inverse problem, which returned the *p*-largest indices of s as a result of a heuristic sensor node selection. In this study, the idea above is straightforwardly extended to the Gramian. A smooth convex objective function approximates Equation (10) as follows:

(13) $$\begin{array}{c} \begin{array}{cc} \; & \begin{array}{cc} \underset{s}{\mathrm{maximize}}\; & \log\det\left(Q\left(s\right)\right)+\kappa \sum_{i=1}^{n}\left(\log\left(s_{i}\right)+\log\left(1-s_{i}\right)\right) \end{array}\\ \; & \begin{array}{cc} \mathrm{subject to}\;\; & s_{i}\in \left(0,\;1\right),\;1{\;}^{⊤}s=p \end{array} \end{array} \end{array}$$

with κ>0, which adjusts the smoothness of the objective function. A Newton step $\delta s\in R^{n}$ is determined by minimizing the second-order approximation of the objective function under a constraint δs=0 as discussed in the Section 10.2 of [50,81], with notation *f* referring to the objective function in Equation (13), as shown below:

(14) $$\begin{array}{cc} \; & \delta s={\left(\nabla^{2}f\right)}^{-1}\left(-\nabla f+\frac{1^{⊤}{\left(\nabla^{2}f\right)}^{-1}\nabla f}{1^{⊤}{\left(\nabla^{2}f\right)}^{-1}1}1\right). \end{array}$$

The first and second derivatives, with respect to the selection weight s, are given by

(15) $$\begin{array}{cc} {\left[\nabla f\right]}_{i} & =c_{i}\left(\sum_{k=0}^{\infty }A^{k}Q^{-1}\left(s\right){\left(A^{⊤}\right)}^{k}\right)c_{i}^{⊤}+\frac{\kappa }{s_{i}}-\frac{\kappa }{1-s_{i}}, \end{array}$$

(16) $$\begin{array}{cc} {\left[\nabla^{2}f\right]}_{i,j} & =-c_{i}H_{j}\left(s\right)c_{i}^{⊤}-\delta_{i,j}\left(\frac{\kappa }{s_{i}^{2}}+\frac{\kappa }{{\left(1-s_{i}\right)}^{2}}\right), \end{array}$$

where $\delta_{i,j}$ is the Kronecker delta, and

(17) $$\begin{array}{c} H_{j}\left(s\right):=\sum_{k=0}^{\infty }\left[\begin{array}{c} A^{k}Q{\left(s\right)}^{-1}W_{\;O}\left(\{j\}\right)Q{\left(s\right)}^{-1}{\left(A^{⊤}\right)}^{k} \end{array}\right] \end{array}$$

is the solution of the following Lyapunov equation;

(18) $$\begin{array}{cc} \; & AXA^{⊤}-X+Q{\left(s\right)}^{-1}W_{\;O}\left(\{j\}\right)Q{\left(s\right)}^{-1}=0. \end{array}$$

The procedure is carried out according to Algorithm 1, where the iteration is completed when the $\ell_{2}$ norm of the solution update falls below the threshold given.
**Algorithm** **1** Newton algorithm for Equation (13)**Input:** $C\in R^{n\times r},\;A\in R^{r\times r},\;p\in N$**Output:** Indices of chosen *p* sensor positions $I_{p}$ Set an initial weight s←1p/n **while** convergence condition not satisfied **do**  Calculate ∇f by Equation (15) and $\nabla^{2}f$ by Equation (16)  Calculate δs by Equation (14)  Obtain step size *t* by backtracking line search  Set s←s+tδs **end while** Return the indices of the *p*-largest components of s as $I_{p}$


One of the most computationally demanding steps of the Newton method in Section 3.1.2 is the inverse of the Hessian in Equation (14), approximately reaching $O(n^{3})$. The rest of this section describes an efficiency improvement for the Newton method of Algorithm 1, as the computational cost was relaxed using small sketches, as in [82,83]. The dimension of the sketched Newton step is $\tilde{n}<n$, where the results for $\tilde{n}/n=0.1$ in [83] showed a drastic reduction in the total computation time for convergence in spite of the increase in the number of iterations. In this study, a sketching matrix $S_{\tilde{n}}\in R^{\tilde{n}\times n}$ is constructed using a permutation matrix for ease of computation, as summarized in the following and in Algorithm 2. The subspace referring to the permutation is randomly assigned from the *n* components, which is indexed by;

(19) $$\begin{array}{cc} \; & I_{\;\tilde{n}}=\left\{i_{1}^{\prime },\;\ldots ,\;i_{\rho }^{\prime },\;\ldots i_{\tilde{n}}^{\prime }\right\}. \end{array}$$

This random selection is biased, according to the selection weight s in the previous iteration. This heuristic leads to a reasonable acceleration of the convergence because the weights of higher weighted sensors in the first few iterations are more likely updated frequently for convergence. In this study, half of the permutation coordinates correspond to the indices of the highest $\rho =\tilde{n}/2$ values of s. The other half of the permutation coordinates were randomly selected from the remaining $n-\tilde{n}/2$ dimensions, and the exploration of sensor selection was further accelerated.

Accordingly, the calculations of the gradient (the first term in Equation (15)) and the Hessian (also the first term in Equation (16)) are simplified to the subspace indexed by $I_{\;\tilde{n}}$. These subsampled derivatives and the derived Newton step are denoted by $\nabla \tilde{f}$, $\nabla^{2}\tilde{f}$, and $\delta \tilde{s}.$ The criterion of the convergence is modified from that in Algorithm 1 due to the randomness, where the algorithm stops if the update size is less than a given threshold in $n/\tilde{n}$ consecutive iterations.
**Algorithm** **2** Customized algorithm of Algorithm 1 (*BRS-Newton*)**Input:** $C\in R^{n\times r},\;A\in R^{r\times r},\;p>0,\;\tilde{n}>0$**Output:** Indices of chosen *p* sensor positions $I_{p}$ Set s←1p/n **while** convergence condition not satisfied **do**  Select $I_{\;\tilde{n}}$ [Equation (19)] and set $S_{\tilde{n}}$  Calculate subsampled derivatives $\nabla \tilde{f}$ and $\nabla^{2}\tilde{f}$  Calculate $\delta \tilde{s}$  Obtain step size *t* by backtracking line search  Set $s\leftarrow s+t\left(S_{\tilde{n}}^{⊤}\delta \tilde{s}\right)$ **end while** Return the indices of the *p*-largest components of s as $I_{p}$


### 3.2. Greedy Algorithms

This type of algorithm sequentially adds the most effective sensor to the subset determined in the previous greedy iterations. The greedy algorithms, therefore, approximate the combinatorial aspect of the original optimization problem, yet preserve the discrete optimization structure. However, even the use of the greedy selection is empirically known to be prohibitive for the system of high-dimensional state vectors and observation vectors; therefore, the accelerating method is often applied [55,56]. Section 3.2.2 provides an approximated form of the greedy selection.

#### 3.2.1. Greedy Selection with Simple Evaluation (*Pure Greedy*)

In the selection of the *q*-th sensor, where 1≤q≤p, the Gramian $W_{\;O}\left(I_{q}\right)$ is obtained from the algebraic Lyapunov Equation (9). The submodularity in the objective function guarantees the quality of greedy solutions [52,53,84], and the derivation of the submodularity of several metrics related to the Gramian is discussed in detail in [66]. This method starts with finding the most effective single sensor; therefore, the determinant can be zero if the observability is not obtained by any single sensor. As mentioned in [66], one may evaluate the objective function for the observable subspace on such occasions. Algorithm 3 starts by decomposing the Gramian for each sensor candidate and identifying its subset $I_{*}$, which results in the highest dimension of the observable subspace, then evaluates the determinant of the decomposed Gramian ${\hat{W}}_{\;O}\left(I_{q-1}\cup \left\{i\right\}\right)$ into the observable space by multiplying their nonzero eigenvalues. Once the full-rank Gramian is achieved by an obtained subset, the greedy algorithm drops the computation of the rank of the candidate Gramian since the rank is monotone-increasing [66].
**Algorithm** **3** Determinant-based greedy algorithm (*pure greedy*)**Input:** $C\in R^{n\times r},\;A\in R^{r\times r},\;p\in N$**Output:** Indices of chosen *p* sensor positions $I_{p}$ $I_{n}\leftarrow \left\{1,\ldots ,n\right\},\;I_{0}\leftarrow \emptyset ,\;$ **for** q=1,…,p **do**  $I_{*}\leftarrow \left\{i:i\in \underset{i\;\in \;I_{n}\;\backslash \;I_{q-1}}{\mathrm{argmax}}\mathrm{rank}W_{\;O}\left(I_{q-1}\cup \left\{i\right\}\right)\right\}$  $i_{q}\leftarrow \underset{i\;\in \;I_{*}}{\mathrm{argmax}}\log\det{\hat{W}}_{\;O}\left(I_{q-1}\cup \left\{i\right\}\right)$  $I_{q}\leftarrow I_{q-1}\cup \left\{i_{q}\right\}$ **end for**


#### 3.2.2. Greedy Selection with Gradient Approximation (*Gradient Greedy* Proposed in This Study)

**Figure 2:**
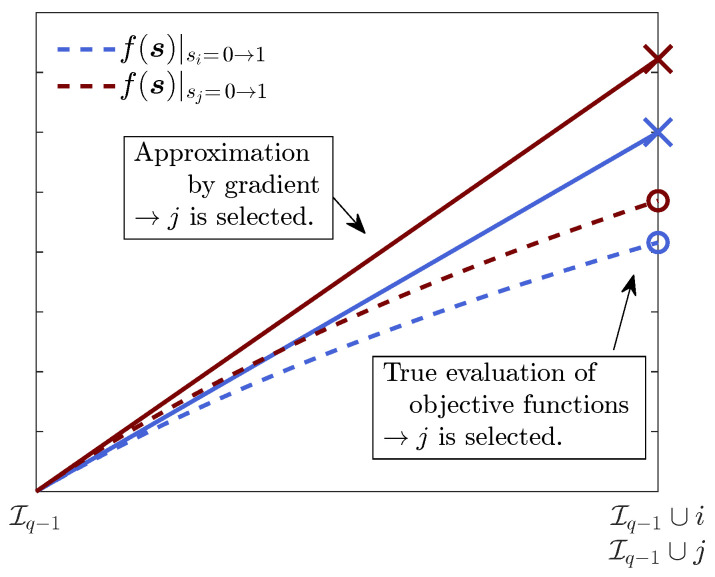
Schematic of the linear approximation of the gradient greedy algorithm for a convex function f(s).

Algorithm 4 proposed in this paper is expected to accelerate the greedy evaluations by the linear approximation of the objective function, which is schematically explained in Figure 2.

The gradient greedy algorithm selects, in the current step *q*, a sensor corresponding to the element of the highest gradient of the objective function f(s) in the prior step. Recall the gradient with respect to the *i*-th sensor candidate of Equation (15);

$$\begin{array}{c} {\left[\nabla \log\det\left(Q\left(s\right)\right)\right]}_{i}=c_{i}\left(\sum_{k=0}^{\infty }A^{k}Q^{-1}\left(s\right){\left(A^{⊤}\right)}^{k}\right)c_{i}^{⊤}, \end{array}$$

where $c_{i}$ is the same notation as used in (13). In the selection of the *q*-th sensor, the *i*-th component of $\nabla \log\det\left(Q\left(s\right)\right)$ is given by replacing $Q\left(s\right)\to W_{\;O}\left(I_{q-1}\right)$ as follows:

(20) $$\begin{array}{c} \lim_{Q\left(s\right)\to W_{\;O}\left(I_{q-1}\right)}{\left[\nabla \log\det\left(Q\left(s\right)\right)\right]}_{i}=c_{i}\left(\sum_{k=0}^{\infty }A^{k}W_{\;O}{\left(I_{q-1}\right)}^{-1}{\left(A^{⊤}\right)}^{k}\right)c_{i}^{⊤}. \end{array}$$

This approximation drops the number of evaluations of the Lyapunov equation and the matrix determinant from the greedy selection, and instead computes inner products of vectors with weighting matrix including the Gramian determined in the previous search. Note that the inverse in Equation (20) is singular when the system is unobservable. A small regularization term $\mathrm{diag}\left[\delta ,\;\delta ,\;\ldots ,\;\delta \right]$ is therefore added to $W_{\;O}\left(I_{q-1}\right)$ to ensure its regularity.
**Algorithm** **4** Determinant-based gradient greedy algorithm (*Gradient greedy*)**Input:** $C\in R^{n\times r},\;A\in R^{r\times r},\;p\in N,\;\delta >0$**Output:** Indices of chosen *p* sensor positions $I_{p}$ $I_{n}\leftarrow \left\{1,\ldots ,n\right\},\;I_{0}\leftarrow \emptyset ,\;$ **for** q=1,…,p **do**  $W_{\;O}\left(I_{q-1}\right)\leftarrow W_{\;O}\left(I_{q-1}\right)+\mathrm{diag}\left[\delta ,\;\delta ,\ldots ,\;\delta \right]\in R^{r\times r}$  Find M *s.t.* $AMA^{⊤}-M+W_{\;O}{\left(I_{q-1}\right)}^{-1}=0$  $i_{q}\leftarrow \underset{i\;\in \;I_{n}\;\backslash \;I_{q-1}}{\mathrm{argmax}}c_{i}Mc_{i}^{⊤}$  $I_{q}\leftarrow I_{q-1}\cup \left\{i_{q}\right\}$  Calculate $W_{\;O}\left(I_{q}\right)$ **end for**


### 3.3. Expected Computational Complexity

This section describes the expected computational complexity of selection methods based on the matrix operations found in textbooks of basic linear algebra. The leading terms, with respect to the system dimension parameters n,p,r, are summarized in Table 1. This also gives the expected overhead term of the run time for each algorithm, while in practice, more compact expressions can be obtained, depending on the libraries for computation employed and the problem structures considered. Readers should also note that the notation of complexity O(◯) conventionally omits the constant factors for simplicity and, therefore, lower-order terms might overwhelm the others in actual computations.

Although the optimal subset $I_{p}$ for Equation (10) can be found by calculating the objective function for all subsets of which the member size is *p*, the computational complexity of this brute-force search will be $O\left(n^{p}r^{3}\right)$ and barely amenable. As for the existing approaches, a naive implementation of the linear convex relaxation method assuming the semidefinite problem (SDP) structure is a simplified form of [67]. The interior point method and path-following iterations should require $\left(O\left(n^{4}\right)+O\left(n^{2}r^{2}\right)+O\left(nr^{3}\right)\right)$ per iteration to construct the Newton direction. This is due to the linear matrix inequality (LMI) constraints, such as the Gramian, being semidefinite, or the selection variable being bounded, while it is known that certain problem structures ameliorate the overall costs ([81], Section 11.8). A simple greedy algorithm (*pure greedy*) requires solving the Lyapunov equation and calculating the determinant of the observability Gramian, of which, the cost is $O\left(r^{3}\right)$, for all *n* sensor candidates and *p* sensor increment iterations. The overall computational complexity is, therefore, $O\left(pnr^{3}\right)$.

The proposed methods will accelerate both of the existing approaches so far by comparing the theoretical complexities. The proposed convex relaxation method (denoted by *approximate convex relaxation*) utilizes the Newton method iterations for the sensor selection. The algorithm requires $O\left(n^{3}\right)$ computations of the inversion of Hessian. Furthermore, Equation (18) will be solved for every j∈{1,2,…,n} for Equation (16), which requires computational complexities of $O\left(nr^{3}\right)$ for solving the algebraic Lyapunov Equation (18) and obtaining the matrix multiplication inside, respectively. The first term in Equation (16) requires $O\left(n^{2}r^{2}\right)$; therefore, the leading terms will be the sum $\left(O\left(n^{3}\right)+O\left(n^{2}r^{2}\right)+O\left(nr^{3}\right)\right)$ per iteration. The sketching matrix, in Algorithm 2, compresses the dimension of the Newton system to $n\to \tilde{n}$, and reduces the costs of the above-mentioned computations by ratio. However, its computational complexity notation does not change. The proposed gradient greedy algorithm (*gradient greedy*) replaces the evaluations of the Lyapunov equation over *n* sensor candidates with inner products of vectors weighted by an r×r matrix. The overall computational complexity will be $O\left(pr^{3}\right)+O\left(pnr^{2}\right)$, where an evaluation of the algebraic Lyapunov equation is taken only *p* times.

## 4. Comparison and Discussion

Numerical examples illustrate the efficiency of the considered algorithms in Section 4. The tested systems, which are represented by $\left(C,A\right)$, are generated by synthetic datasets of random numbers and real-world counterparts of large-scale measurements. As used in Section 2, the systems of interest are in discrete-time LTI forms. The entire computation was conducted on MATLAB R2022a, and CVX 2.2 [85,86]; the MOSEK solver was used for the SDP-based selection, as noted in Appendix A. The dlyap function was used for the solutions of the discrete-time Lyapunov equations, such as Equations (9) and (18), adopting the subroutine libraries from the Subroutine Library in Control Theory (SLICOT) [87,88,89,90]. It should also be noted that there is another approach to solving the equation, such as [91]. The MATLAB programs are available through the GitHub repository of the present authors [92].

### 4.1. Results of the Randomly Generated System

**Figure 3:**
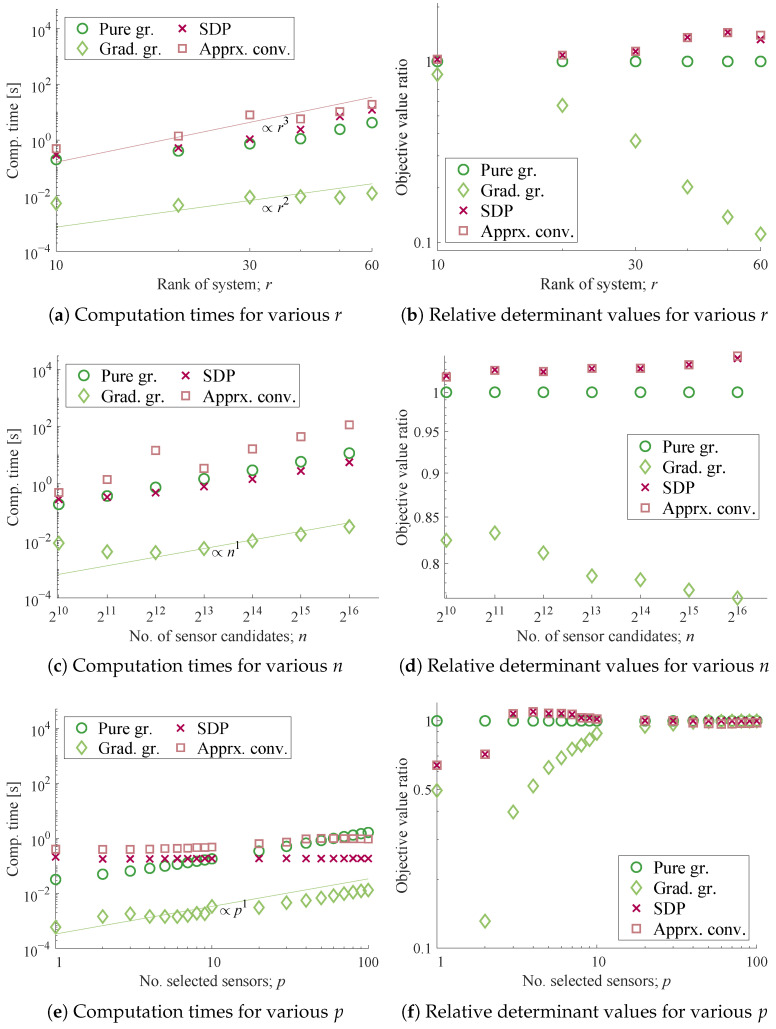
Computation times and optimization results for randomly generated systems, for (**a**,**b**) varying the number of state variables *r*, i.e., the size of A matrix (average of 100 times trial, n=1024,p=10); (**c**,**d**) varying the number of sensor candidates *n*, i.e., the rows of C matrix (average of 20 times trial, p=10,r=10); (**e**,**f**) varying the number of sensors selected *p*, (average of 100 times trial, r=10,n=1024).

The characteristics of the sensor selection methods are investigated for different sets of system dimension parameters (n,r,p) by varying one parameter while fixing the others. The abscissa of Figure 3a,b is the rank r∈{10,20,…,60}, which is the dimension of the reduced state variable vector and, thus, of the Gramian. In Figure 3c,d, $n\in \{2^{10},2^{11},\ldots ,2^{16}\}$ denote the sizes of node members comprising the original measurements, and accordingly the number of sensor candidates. In Figure 3e,f, p∈{1,2,…,10,20,…,100} represents the varying number of sensors selected.

The problem setting for the system of random numbers is found in [39]. First, the conjugate complex numbers, of which the real parts are negative, are assigned to the eigenvalues of a damping continuous-time system matrix $\hat{A}$. A discrete-time system matrix can be obtained by $A=\exp(\hat{A}\Delta t)$, which is stable and full-rank, whereas Δt is the time step of the discrete system. The observation matrix C is a column-orthogonal matrix generated by the singular value decomposition for a matrix of Gaussian random numbers of appropriate dimensions. Sensors up to *p* are selected using the algorithms presented in the previous section, and the objective function, $\log\det W_{O}\left(I_{p}\right)$, is calculated for each selected subset. Figure 3 shows the performance of the selection methods applied to the system of random numbers.

Figure 3a,c,e illustrate the total computation time of each algorithm in the tests, where the gradient greedy method is the least time-consuming, followed by the pure greedy and the SDP-based methods, which produce similar results. Unfortunately, the proposed approximate convex relaxation method took the longest time to solve the optimization in almost all of the conditions tested despite its acceleration owing to the customized randomization. However, it is also expected that the computation time of the SDP-based method and the pure greedy selection will exceed the others for even larger *r* and *p*, respectively.

The empirical orders of the computation time with regard to each parameter are analyzed herein. The growth rates of the computation time of each algorithm are evaluated by solely changing the system dimension parameters *r*, *n*, and *p*, as summarized in Table 2, based on the results shown in Figure 3a,c,e. First, the gradient greedy method ran in time proportional to *n*, but it is not clear regarding *r*. Since the number of sensor candidates, *n*, is much larger than *r* in the first experiment, the term with $r^{3}$ is not significant for the gradient greedy method. The increase against *p* is on the order of unity, which is clearly reasonable. Second, the empirical orders of the pure greedy method when solely changing *r*, *n*, and *p* are $r^{3}$, *n*, and *p*, respectively, which agrees with the expected leading order. One can see a term with $r^{3}$ becomes dominant as *r* grows in Figure 3a. Meanwhile, the estimated orders of the SDP-based method when solely changing *r* and *n* are $r^{\left[4\right]}$ and *n*, while those of the approximate convex relaxation method are $r^{\left[3\right]}$ and $n^{\left[2\right]}$, respectively. Here, let the notation $\left[\;j\;\right]$ stand for (despite an obvious abuse) a real number *x* bounded by j−1 and *j* for an arbitrary positive integer *j*. These noninteger orders may be due to the optimized arithmetic employed in the software, such as the Strassen-like algorithm [93]. The dependency regarding *p* was not clear in the SDP-based method, while a slight increase was observed for the approximate convex relaxation method.

The interesting aspect is that the dominating order of the SDP-based method with respect to *n* was not $n^{4}$, which was initially expected but is approximately proportional to *n*. This is perhaps because the constraint posed as $0\leq s_{i}\leq 1$ in Appendix A was simplified in the tested implementation as a mere diagonal block of the semidefinite linear matrix inequality (LMI), and the MOSEK solver should have taken advantage of its structure during the Newton step calculation, despite the large *n* assumed. It should also be noted that this efficacy was not observed for other solvers, such as the SDPT3, although the CVX parser does not seem to change its output. Nonetheless, the complexity of solving the SDP would be enormous, as expected, if the LMI included such a semidefinite relaxation [61,94] of the selection variable vector s, such as $ss^{⊤}$ being a semidefinite matrix. This agrees well with the observations from the experiment for the SDP-based method regarding *r*, where the LMI of the dimension of r×r is included.

In addition to the computation time per step, the iteration numbers before convergence, as shown in Table 3, also illustrate the computationally friendly features for the high-dimensional problems. The iteration numbers of the SDP-based and the approximate convex relaxation methods do not increase significantly as *r*, *n*, and *p* individually increase. Interestingly, the iteration numbers of these convex relaxation methods change in a different fashion. That of the SDP-based method slightly increases with increases in *n* and *p*, and slightly decreases with an increase in *r*, while that of the approximate convex relaxation shows opposite results. This leads to similar growth rates against *r* between these two methods, as shown in Figure 3a, where the empirical computational complexity is less than $O(r^{4})$ for the SDP-based method and more than $O(r^{3})$ for the approximate convex relaxation method. Moreover, Figure 3c shows that this difference leads to growth rates that are slightly larger than O(n) for the SDP-based method and smaller than $O(n^{3})$ for the approximate convex relaxation method, against *n*. This unexpected efficiency of the SDP-based method leads to better scalability with respect to *n* over the approximate convex relaxation, which is the other convex relaxation method. With regard to the increase in *p*, the iteration numbers of the SDP-based method increased only slightly, while those of the approximate convex relaxation method exhibited an increase around p=20, corresponding to the increase in the total run time in Figure 3e.

As shown in Figure 3b,d,f, almost the same objective values were obtained by the SDP-based and the approximate convex relaxation methods, which implies a good agreement between the solutions of these relaxation methods. They yielded better or comparable objective function values as compared to the greedy methods, which were up to twice as high, except for p=1,2 cases. Those obtained by the gradient greedy method, on the other hand, give an inferior impression as a selection method as compared with the other methods for p≤r cases. This is possibly due to the difficulty in ensuring the observability with a limited number of sensors, especially r>p, which should lead to an unstable calculation of the gradient Equation (20). The gradient greedy algorithm is concluded to have poor performance in achieving observability, especially when the convex objective function is hardly approximated by its linear tangent due to large *r*.

The discussion above illustrates that the use of the SDP-based method seems to be the most favorable among the compared methods in this experiment. The use of the pure greedy method is also a reasonable choice due to its shorter computation time, straightforward implementation, and reasonable performance, which returns half the objective function value of the convex relaxation methods in the experiments.

### 4.2. Results for Data-Driven System Derived from Real-World Experiment

**Figure 4:**
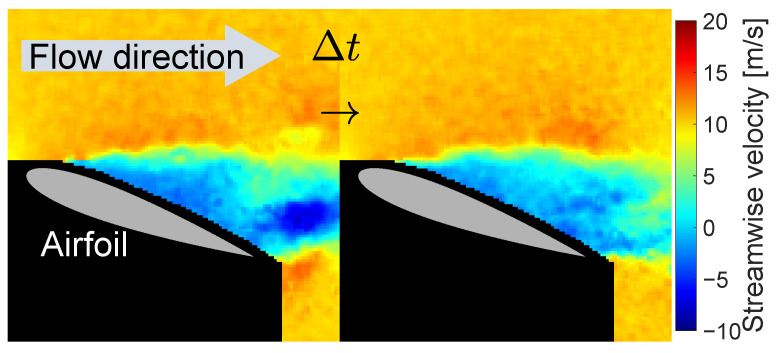
Visualized flow around NACA0015 airfoil [95,96]. Streamwise components in a time-series measurement are shown and used in the demonstration to construct the reduced-order state variables Equation (21). (Δt≈0.01 s in this figure).

An example of a practical application was conducted using the experimental dataset of flow velocity distribution around an airfoil. The data used herein are found in [95,96], which were previously acquired by the particle image velocimetry (PIV) in a wind tunnel. A brief description of the experiment is shown in Table 4; refer to the original article by the authors for more specific information. The snapshots taken in the experiment quantify velocity vectors that span the visualized plane, i.e., two components on a two-dimensional grid of n=9353 points, as depicted in Figure 4.

Only the streamwise components (the direction shown by the arrow in Figure 4) are used, and the ensemble averages over *m* snapshots are subtracted at each measurement location; that is, averages for each pixel of the calculated velocity image.

As attempted in Section 4.1, a linear representation $\left(C,A\right)$ is derived first. The data-driven system identification procedure is based on the modeling method of [30,78]. Here, the snapshots of the velocity field are reshaped to (n×1)-dimensional *m* vectors, $y_{1},\;\ldots ,\;y_{m}$, then the data matrix is defined by $Y:=\left[y_{1},\;\ldots ,\;y_{m}\right]\in R^{n\times m}$. The proper orthogonal decomposition (POD) is then adopted and the data are projected onto the subspace of the leading *r* POD modes [37], resulting in $Y\approx U\Sigma V^{⊤}$ ($U\in R^{n\times r},\Sigma \in R^{r\times r},V\in R^{m\times r}$, respectively). The measurement matrix C is defined by U, which consists of *r* left singular vectors related to the largest *r* singular values. These left singular vectors illustrate the dominant spatial coherent structures, while the right vectors represent temporal coherence, and the singular values are the amplitudes of these modes. The use of POD in our study is intended for a more fundamental discussion based on a linear system representation where arbitrary-order low-rank systems are derived from high-dimensional measurement data.

The *r*-dimensional state variable vector x is given by

(21) $$\begin{array}{c} \left[x_{1},\;\ldots ,\;x_{m}\right]:=\Sigma V^{⊤}\in R^{r\times m}. \end{array}$$

After introducing $X_{1}=\left[x_{1},\;\ldots ,\;x_{m-1}\right]$ and $X_{2}=\left[x_{2},\;\ldots ,\;x_{m}\right]$, the system matrix is then expressed using linear least squares via the pseudo-inverse operation $A=X_{2}{\left(X_{1}\right)}^{†}$. The system matrix A is manipulated to ensure that its eigenvalues are less than unity and, therefore, the considered systems are stable.

As discussed in Section 4.1, the comparisons are illustrated using the objective function values obtained. The sensor candidate set corresponds to each pixel obtained by the experimental visualization and, thus, its size is fixed to n=9353. The state variable vector is set to have r=10,…,60 components as a result of the order reduction at different truncation thresholds, while *p* is fixed to 20. Moreover, the results with respect to various *p* values are also provided, where p=1,2,…,10,20,…,100 with r=10 being fixed. The original dataset consisting of the 10,000 snapshots is divided into m=2000 consecutive snapshots and, therefore, the following results are the five-fold average.

**Figure 5:**
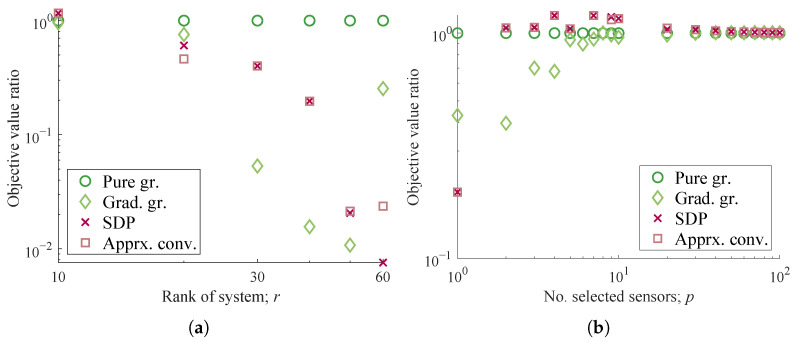
Optimization results for a system based on the experimental data of fluid dynamics. (**a**) Relative determinant values for various *r* (p=20,n=9353). (**b**) Relative determinant values for various *p* (r=10,n=9353).

The values of the objective function show similar trends to Figure 3b,f for the gradient greedy algorithm, while a large degradation is observed in the convex relaxation methods for r>p. Surprisingly, those of the convex relaxation methods are 2–100 times lower than that of the pure greedy method. This trend is also partially observed for the p=1 case in Figure 5b, where the pure greedy selection corresponds to the true optimum for this configuration by definition. This clear degradation in the convex relaxations has not yet been explained in this study and may be due to the ill condition of the low-order approximation or the numerical error. A similar discussion was conducted for the previous study [66]. The cause should be investigated in detail for a practical application with the real-world data in a future study.

The results above, together with those in the previous subsection, illustrate that the pure greedy method shows a more stable performance, while the convex relaxation methods show wide variations in their performance. Therefore, the pure greedy method can be considered the most appropriate method, taking into account the computation time. It should be noted that the present study also shows the prominent performance of the SDP-based method in terms of the objective function and the computation time in the randomly generated, general problem settings. As long as the additional computation time is acceptable, the present authors recommend trying both the pure greedy and the SDP-based methods and to use a better sensor set when selecting the sensors based on the observability Gramian.

### 4.3. Results for Data-Driven System Derived from Weather Observation

**Figure 6:**
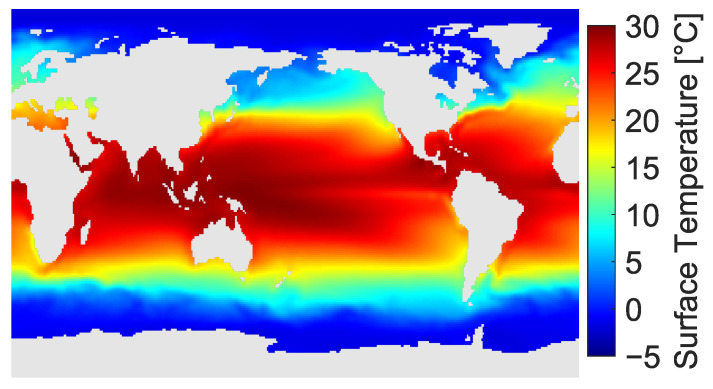
Mean value distribution of the global sea surface temperature (from 31 December 1989 to 29 January 2023).

A data-driven linear model is obtained from the time-series data of the global sea surface temperature (SST), as conducted in Section 4.2. Table 5 and Figure 6 briefly describe the conditions of processing for the SST dataset. The satellite observation, along with in situ correction composites, interpolated the distribution of SST, and the weekly mean distribution was stacked in the data matrix as a time-series measurement spanning from 1989 to 2023. The data matrix consists of 1727 snapshots of 44,219 spatial components, which correspond to the number of columns and rows, respectively. The sensor selection application to the SST dataset is a common benchmark, as found in [2,40,41,63,97,98].

The linear model was constructed in the same data-driven manner as previously explained in Section 4.2. Moreover, with a dimension set of the system given by *r* = 10, *n* = 44,219 and p=10, the evaluation was conducted as in the previous section by varying *r* and *p* solely as r=10,20,…,60, and p=1,2,…,10,20,…,100, respectively. The whole duration was used for the construction of the reduced-order model in this example.

**Figure 7:**
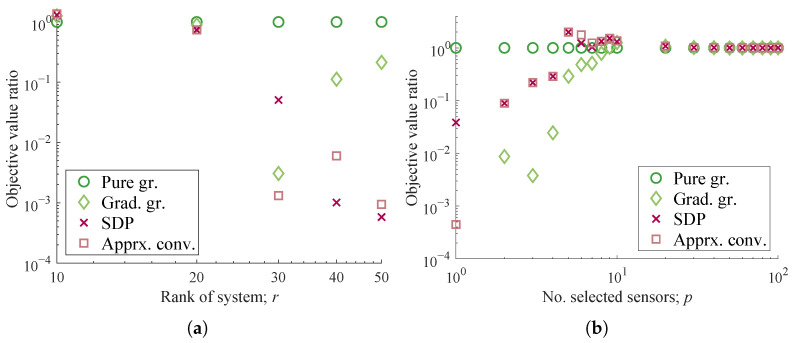
Optimization results for a system based on the experimental data. (**a**) Relative determinant values for various *r* (*p* = 10, *n* = 44,219). (**b**) Relative determinant values for various *p* (*r* = 10, *n* = 44,219).

The sensor selection results in Figure 7a,b support the observations of the real-world examples of PIV measurements in Section 4.2, as the optimization measures of the SDP-based method and the approximate relaxation method similarly returned deteriorated results as compared with those of the pure-greedy method at p≤r.

## 5. Conclusions

This research investigates efficient algorithms that give beneficial sensor positions based on the observability Gramian of a discrete-time linear dynamical system, and are essential to realizing effective state estimation and control. The selection methods were characterized using synthetic numerical examples and a data-driven system representation of a real-world dataset. An analysis of the increase in computation time with respect to the system dimension parameters showed the effectiveness of each selection method, along with comparisons of their optimization performance.

This study offers two novel approaches for sensor selection: an approximate convex relaxation solved by a customized Newton method and a linear approximation of a pure greedy evaluation. A comparison was conducted against conventional methods, including a convex relaxation to the semidefinite programming and the pure greedy method. The proposed gradient greedy method achieved a moderate solution with orders-of-magnitude speedups compared to the other methods when the dimensions of the system’s state variables did not exceed the number of sensors deployed. Meanwhile, this method was found to be incapable of ensuring the observability of the larger dimensions of the state variable. The convex relaxation methods, including the SDP-based and the approximate convex relaxation methods, achieved better solutions than the greedy methods in synthetic data, while the real-world example discloses their unstable optimization results, especially for a large-scale system. In less observable situations, the pure greedy selection is supposed to be the most reliable choice in terms of the optimization metric obtained.

## Figures and Tables

**Table 1:** Selection algorithms for each relaxed problem and the expected arithmetic complexity order based on the basic matrix operations. **Several approaches** are proposed in the presented paper.

Problem	Algorithm	Expected Complexity
Linear relaxation SDP [67]	Path-following method	$\left(O\left(n^{4}\right)+O\left(n^{2}r^{2}\right)+O\left(nr^{3}\right)\right)$/iter.
Approximate convex relaxation	**BRS-Newton** [Algorithm 2]	$\left(O\left(n^{3}\right)+O\left(n^{2}r^{2}\right)+O\left(nr^{3}\right)\right)$/iter.
Greedy [66]	Pure greedy [Algorithm 3]	$O(pnr^{3})$
Greedy	**Gradient greedy** [Algorithm 4]	$O\left(pnr^{2}\right)+O\left(pr^{3}\right)$

**Table 2:** Practical orders of the computation times of selection methods, investigated with respect to each parameter individually.

Selection Method	*r*	*n*	*p*
*SDP*	$r^{\left[4\right]}$	*n*	$p^{0}$
*Approx. conv.* [Algorithm 2]	$r^{\left[3\right]}$	$n^{\left[2\right]}$	$p^{\left[1\right]}$
*Pure greedy* [Algorithm 3]	$r^{3}$	*n*	$p^{1}$
*Gradient greedy* [Algorithm 4]	N/A	*n*	$p^{1}$

**Table 3:** Iteration numbers of the convex relaxation approaches, investigated with respect to each parameter individually. Averages are rounded to integers.

(a) Various *r* for Randomly Generated Systems, Average of 100 Trials							
*r*	10	20	30	40	50	60	
*SDP*	18	13	11	12	12	12	
*Approx. conv.*	203	293	348	373	380	388	
(**b**) Various *n* for randomly generated systems, average of 20 trials							
*n*	$2^{10}$	$2^{11}$	$2^{12}$	$2^{13}$	$2^{14}$	$2^{15}$	$2^{16}$
*SDP*	18	21	23	27	28	31	33
*Approx. conv.*	209	167	147	141	139	139	141
(**c**) Various *p* for randomly generated systems, average of 100 trials							
*p*	1	2	4	8	10	20	40
*SDP*	14	13	15	17	18	18	17
*Approx. conv.*	171	168	172	198	206	273	411

**Table 4:** Brief descriptions of PIV data [95,96].

(a) Wind Tunnel Experiment	
Airfoil	NACA0015 (Chord length 100 mm)
Wind tunnel	Recirculating low-speed wind tunnel
Flow speed	10 m/s
Angle of attack	$18^{{}^{\circ}}$
(**b**) Acquisition condition of velocity distribution	
Sampling rate	5000 Hz
Spatial sample	9353 points
Snapshot sample	2000 snapshots × 5 sets

**Table 5:** Brief descriptions of sea surface temperature data [99,100].

Data type	Weekly means from 31 December 1989 to 29 January 2023
Grid scale	1.0 degree lat × 1.0-degree long grid (180 × 360)
Spatial sample	44,219
Temporal sample	1727

## Data Availability

The MATLAB codes used for this manuscript are found in the GitHub repository [92]. The real-world experimental data used in the results section are also in another repository [96].

## Appendix A. CVX Implementation for Equation (12)

Thanks to the parser CVX [85,86], the SDP formulation defined by Equation (12) is easily implemented in MATLAB and solved by using the MOSEK solver in [67] and in our case. The actual implementation is shown below, and MATLAB codes are provided in [92].

     [r,~] = size(A);

     [n,~] = size(C);

     cvx_solver(’MOSEK’)

     cvx_begin sdp

        variable z(n) nonnegative

        variable X(r,r) symmetric semidefinite

        maximize( det_rootn(X) )

        subject to

            % matrix inequality

            A’*X*A-X+C’*(repmat(z,1,r).*C) >= zeros(r,r);

            % bounds selection variables

            z <= 1;

            sum(z) == p;

     cvx_end
